# Esophageal variceal ligation for hemostasis of acute variceal bleeding: efficacy and safety

**DOI:** 10.11604/pamj.2013.14.95.1847

**Published:** 2013-03-10

**Authors:** Mounia Lahbabi, Mounia Elyousfi, Nouredine Aqodad, Mohammed Elabkari, Ihssane Mellouki, Sidi Adil Ibrahimi, Dafr Allah Benajah

**Affiliations:** 1 Department of Hepato Gastroenterology Hassan II University Hospital Fez, Morocco

**Keywords:** Esophageal variceal ligation, variceal bleeding, endoscopy, rebleeding, complications

## Abstract

**Introduction:**

Endoscopic variceal ligation is widely accepted as the optimum endoscopic treatment for esophageal variceal hemorrhage. In Morocco, there are no data regarding the efficacy of this technique. Our aim was to evaluate the effectiveness and safety of endoscopic variceal ligation in the management of oesophageal variceal bleeding in cirrhosis in a located population in Morocco.

**Methods:**

Via a retrospective study over 118 months (December 2001- October 2011), cirrhotic patients with endoscopically proven esophageal variceal hemorrhage were treated by endoscopic variceal ligation. We studied the rate of haemostasis, rebleeding, complications and mortality.

**Results:**

360 cirrhotic patients were included and 378 haemostatic variceal ligations were performed. Primary haemostasis was obtained in 96.5 % (N=365) of cases. Thirty three patients (8.7%) bled during follow-up. The rate of minor complications was 15.3 % (N=58). Retrosternal pain, fever, dysphagia and Overtube's migration developed in 8.4 % (N=32); 2.6 % (N=10); 3,7 % (N=14) and 0.5 % (N=2) of the patients respectively. Severity of these complications was mild and transient. The rate of oesophageal ulcers was 5 % (N=19), while the mortality rate by haemorrhage was 5 % (N=18).

**Conclusion:**

Our data showed that band ligation is an effective and safe treatment modality of esophageal variceal bleeding with low rates of rebleeding and complications.

## Introduction

Esophageal variceal bleeding is one of the most serious complications of portal hypertension [1], and represents a leading cause of death in patients with cirrhosis [1]. Each bleeding episode is associated with a 30% mortality rate [2]. Endoscopic band ligation (EBL) is generally accepted as the treatment of choice for bleeding from esophageal varices. It has shown good results in terms of the control of the active bleeding, with few untoward effects [3]. Despite this, there is no evidence how to monitor patients after EBL, and there are no data regarding the efficacy and safety of this technique in our country. Therefore, the aim of this study was to evaluate the effectiveness and safety of EBL in the management of oesophageal variceal bleeding and to analyze outcome of bleeding complications after EBL in a located population in Moroccan cirrhotic patients.

## Methods

This retrospective study was conducted in the Department of Gastroenterology and Hepatology of Hassan II University Hospital Fez, Morocco, from December 2001 to October 2011. Adult cirrhotic patients with an episode of upper gastrointestinal bleeding were admitted to the emergency service, and underwent endoscopy as soon as they had been resuscitated. Vasoactive drugs (somatostatin) were started as soon as a variceal bleeding was suspected and maintained afterwards for 2-5 days. Patients with endoscopically proven esophageal variceal hemorrhage were treated by endoscopic variceal ligation if they had active variceal bleeding at endoscopy (spurting or oozing from esophageal varices); or if they had no bleeding varices but evidence of blood with no other potential source of gastrointestinal bleeding. Patients with liver cancer or other malignancy were excluded from the study. Cirrhosis was documented either on the basis of a liver biopsy performed during a previous admission or typical laboratory, clinical and ultrasonographic findings. All patients were hospitalized until drop-off of all ligation bands, proven by endoscopy. Hemostasis was defined as control of bleeding during the first 24 hours after starting EVL [4]. Data were analyzed by the Epi Info 2000. Quantitative data were expressed as means (±SD) or as medians. The Kaplan-Meier method was used to estimate the rates of survival without bleeding. The study protocol was approved by the ethics institutional review board of our hospital.

## Results

**Figure 1:**
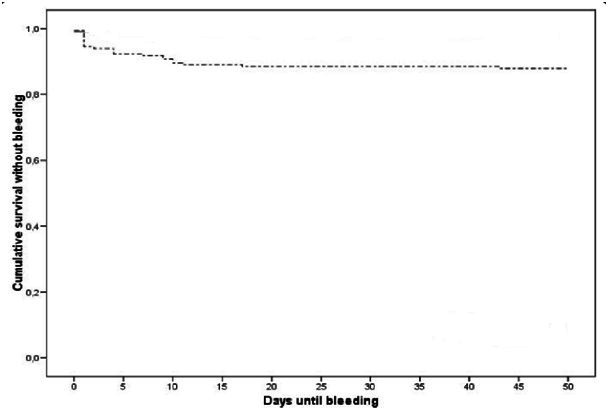
Kaplan-Meier estimation of cumulative survival without hemorrhage for emergency EBL. Ninety one percent of bleeding events occurred within 11 days after treatment. Most rebleedings 75.7% were observed within the first 4 days

Three hundred sixty cirrhotic patients were included in the study, and 378 hemostatic variceal ligations were performed. Clinical and endoscopic data of patients are presented in Table 1. The success rate of hemostasis achieved by EBL was 96.5 % (N=365). The rate of minor complications was 15.3 % (N=58). Major side effects occurred in 19 patients (5%). Complications related to EBL are shown in Table 2. Thirty three patients (8.7%) bled during follow-up. The number of bleeding events from ligation ulcers and variceal rebleeding was 19 (5%) and 13 (3.4%) respectively. In one case, it was not possible to retrospectively evaluate the exact source of bleeding from the endoscopic records. Ninety one percent (30/33) of rebleeding events occurred within 11 days after treatment, most of them 75.7% (25/33) were observed within the first 4 days (Figure 1). Eradication of varices was achieved in 89 % (N=320) of patients, with 3 ± 1.99 (range 1-6) endoscopy sessions. Overall, the mortality rate was 10.2% (N=37). Causes of death are shown in Table 3.

**Table 1:** Clinical and endoscopic characteristics of patients included in the study

Characteristics	N (%)
**Age ± SD (Years)**	52.8 ±7 (16 – 89 years)
**Sex ratio (male/female)**	2.6
**Medical comorbidity**	
Portal hypertension	186 (51.6%)
Diabetes	12 (3.3%)
Compensated heart disease	3 (0.8%)
Arterial hypertension	10 (2.7%)
Chronic renal failure	2 (0.5%)
**Etiology of cirrhosis**	
Viral hepatitis C	211 (58.6 %)
Viral hepatitis B	91 (25.3 %)
Viral hepatitis C and B	7 (1.9 %)
Alcoholic cirrhosis	2 (0.5 %)
Cryptogenic cirrhosis	49 (13.6 %)
**Hemodynamically stable**	308 (85.6%)
**Child-Pugh's score**	
A (%)	131 (36.4%)
B (%)	129 (35.8%)
C (%)	100 (27.8%)
**Esophageal varices**	
Grade II/ Grade III	183 (50.8%)/177 (49.2%)
**Active bleeding at endoscopy (spurting or oozing)**	149 (41.3%)
**Recent bleeding stigmata**	145 (40.3%)
**Propranolol**	26.6% (N=96)

**Table 2:** Complications related to endoscopic variceal ligation

Complications of ligation of esophageal varices	N (%)
**Minors complications**	
Retrosternal pain	32 (8.4%)
Dysphagia	14 (3.7%)
Fever	10 (2.6%)
Overtube's migration	2 (0.5%)
**Majors complications**	
Esophageal ulcers	19 (5%)
Aspiration pneumonia	0
Bacterial peritonitis	0
Empyema or sepsis	0

**Table 3:** Causes of death (N=37)

Causes of death	N (%)
Death of procedure	5 (13.5%)
Death of rebleeding	13 (35%)
Death of spontaneous ascites infection	10 (27%)
Death of liver failure	7 (19%)
Death of non-liver related cause	2 (5.4%)

## Discussion

In this study, EBL controlled bleeding in 96.5 % of patients; whereas therapeutic success were estimated between 86% and 100% in other studies [5]. As a result, EVL lacks adverse systemic effects. Complications associated with the procedure include dysphagia, esophageal strictures, and transient chest discomfort [5]. In our study the rate of minor complications was 15.3 %. Retrosternal pain, fever, dysphagia and Overtube's migration developed in 8.4 %; 2.6%; 3.7 % and 0,5 % of the patients respectively. Altintas et al had reported respective rates of chest pain, fever and dysphagia in 23.8%, 23.8 % and 4.76% of patients [6]. We documented an overall bleeding rate after EBL of 8.7 %. This rate is consistent with those reported in other studies (4 to 36%) [7]. The rate of rebleeding events from ligation ulcers was 5%. In other published studies, this value has been reported as 5.4% to 14.2% in patients who underwent the procedure for control of acute variceal hemorrhage [8]. In this study 91% of rebleeding events after emergency ligation occurred within 11 days after treatment.

## Conclusion

Our study confirms former reports that have demonstrated the safety and effectiveness of EBL for treatment of acute esophageal variceal bleeding. We found that 91% of bleeding events occurred within 11 days after treatment. Therefore, we propose to keep patients who have undergone EBL for treatment of acute variceal bleeding under medical surveillance for at least 11 days.
